# Publishing in open access journals

**DOI:** 10.1186/s13244-024-01794-6

**Published:** 2024-08-26

**Authors:** Emilio Quaia, Chiara Zanon, Alberto Vieira, Christian Loewe, Luis Marti-Bonmatí

**Affiliations:** 1 Department of Radiology, University of Padova, Via Giustiniani 2, 35128 Padova, Italy; 2 Radiology Department at CUF Hospital Porto and Invited Assistant at Porto School of Medicine–Porto University (FMUP), Porto, Portugal; 3 Division of Cardiovascular and Interventional Radiology, Department of Bioimaging and Image-Guided Therapy, Medical University Wien, Wien, Austria; 4 Medical Imaging Department and Biomedical Imaging Research Group at Hospital Universitario y Politécnico La Fe and Health Research Institute, Valencia, Spain

Access to scientific publishing has traditionally been based on subscription-based journal models. The growing use of the internet led to instant access to some scientific manuscripts, with the immediate availability of a huge number of scientific papers in special situations (i.e., individual or institutional subscribers). Open access (OA) introduces a novel approach to publication, offering unrestricted, free availability of research papers, combined with the rights to use them with adequate recognition and without any need for specific journal subscriptions [1]. There are five main types of open access journals, based on different levels of accessibility [2]:

- Gold OA: Articles are immediately accessible on the publisher’s website with costs typically covered by article processing charges paid by authors or their institutions.
- Green OA (self-archiving): Authors deposit versions of their work (preprint, postprint, or published) in a public repository, free of charge, sometimes after an embargo period as per journal policies.
- Hybrid OA: In traditional subscription journals, authors can opt to make their articles open access by paying a fee, allowing for a mix of open access and subscription-based articles.
- Diamond/Platinum OA: Articles are freely accessible without any publication charges to authors, funded instead by institutions, societies, or grants, thereby removing financial barriers for authors.
- Bronze OA: Articles are temporarily free to read on the publisher’s website without an open access license, often as part of promotional access.

Black OA corresponds to illegal behavior because, despite the article not being openly licensed, it is still shared by illicit services offering OA to scientific publications [3].

Among these variants, green OA appears interesting because authors do not change their publication habits and continue to publish in specific journals, with the possibility of self-archiving their accepted or published papers in an institutional repository under agreement with editorial copyright. Green OA presents two variants: the green published research papers available in their final version and the green accepted variant—research papers available in the accepted version after peer review and consequently different from the official published version, both in the paginated and edited editions, but not in the scientific content. Unfortunately, even green OA editors may impose specific restrictions on the availability of research papers, which cannot be immediately made available on the web unless specific tools, such as unpaywalls, are installed [4].

A scientific journal may even offer hybrid OA (also called red OA), where the processing fee, which is often higher than that of a regular OA journal, is paid for specific scientific articles. Hybrid OA is often criticized for representing a double gain, both from official subscriptions and specific paper OA costs for scientific editors [5]. In this review, we will focus on Gold open access, which is the one provided in open access journals such as *Insights into Imaging*.

A critical point to consider is that accessibility and peer review are completely different aspects. Journals can offer OA to readers, but the reviewing process has to be clearly defined. OA, as employed by *Insights into Imaging*, undergoes a thorough peer review process involving multiple reviewers. This iterative evaluation enhances manuscripts’ quality significantly.

Why is OA so successful? OA journals achieve success by enhancing the dissemination of paper content, increasing citation rates, and accelerating academic recognition as well as clinical impact. Most research and innovation funders require that the obtained results be published as OA, with the intent of increasing the availability of the outputs from the research they funded.

As a global trend, there is a growing number of OA publications. Most journals offer this possibility if authors are willing to pay for the article processing charges (APC). Other journals provide only open access, and the APC can be waived in special situations such as authors from low-income countries or papers submitted after invitation. Presently, there are more than 15,500 fully OA journals, and this number is continuously increasing. An updated list of OA journal names can be found in the Directory of Open Access Journals (DOAJ) [6] for all disciplines.

In the research community, OA publications are often part of a national mandate. A mandate corresponds to those requirements set by a governing body, funding agency, or other organization stipulating how research should be conducted, disseminated, or made accessible. In Europe, many OA mandates are widely covered by the Transformative Agreement. These contracts, also referred to as transitional or “read and publish” agreements, are negotiated between institutions and publishers that transform the business model underlying scholarly publishing toward a fully OA model [7]. Japan, Australia, and New Zealand are almost completely covered by the Transformative Agreement [8].

A further initiative that should be mentioned is DEAL, under the leadership of the German Rectors’ Conference, which supports access to scientific papers and covers the article processing charge (APC) for OA publications for researchers affiliated with German institutions [9].

The European Commission strongly supports Gold OA to scientific publications and data, reflecting a commitment to making research results more accessible and promoting better and more efficient science and innovation [10]. The Commission’s OA policies are integrated into its major research and innovation funding programs, such as Horizon 2020 and Horizon Europe. Under these programs, all peer-reviewed scientific publications resulting from funded projects must be freely accessible. This is mandated to ensure that the public and the research community can benefit from OA to scientific outputs. Furthermore, the Commission has developed the Open Research Europe platform, offering a direct publishing route for researchers to share their results widely and immediately, with no costs for the beneficiaries of Horizon programs. This platform exemplifies the Commission’s move toward full open access by providing an immediate, visible, and comprehensive open access venue [10].

The USA is fostering the OA policy with its commitment to making all federally funded research OA by the end of 2025. The White House Office of Science and Technology Policy (OSTP) states the following: (1) The goal of the memo is to provide free, immediate (without embargo), and equitable access to federally funded research. (2) Applies to all federal agencies, including those that fund Humanities and Social Sciences research. (3) Applies to peer-reviewed publications and underlying scientific data [11].

The Chinese policy requests that institutions “promote the development of open science” and the “author pays” model is leading in China. The term “author pays” reflects the shift in the cost of publishing from the reader to the author, and the authors’ funding bodies are expected to cover these costs on their behalf. The Wellcome Trust made a conservative estimate of the cost of publishing an OA article to be between US$500–US$2500, depending on the journal’s level of selectivity [12].

Publications on OA have several advantages. First, research papers are freely accessible on the web and generally have a shorter publication time than other journals. Often, OA publications imply research data availability with increased transparency. Authors increase their visibility with OA publications because more people can see and download research in a true democratic endeavor, and new readers and researchers from outside the established research environment (e.g., from developing countries) may read research papers [13]. Second, OA journals often present high impact factors and provide higher public engagement with alternative metrics such as the number of downloads and Altmetrics corresponding to the journal impact based on diverse online research outputs, such as social media, online news media, online reference managers based on paper viewing, downloading, discussing, saving, and citing on different platforms [14]. Third, in OA, authors remain copyright owners without the need for copyright transfers. OA could provide better quality assurance because research papers may be viewed and assessed by a wider readership, with a consequent reduced potential for plagiarism. Fourth, an OA research paper has the best conditions for being able to participate in interdisciplinary research to carry out collaborative research on a global scale and potentially be more cited [15]. Several studies have shown that OA articles are viewed and cited more often than articles published in subscription-based scientific journals [16], although there are significant disciplinary and platform-specific differences in the OA advantage [17]. Moreover, funding bodies and associations require OA publications with increasing frequency to increase the visibility of research paid through voluntary or national public funding. Every year, many journals convert from closed access to OA, generally with positive effects in terms of citations, publication output, and impact factors [18].

However, OA also has certain disadvantages. First, OA is a paid service, and not all authors can face publication expenses, namely the APC, which may be paid by the authors or by the institutions. Second, not all research is open due to the General Data Protection Regulation (GPDR) and copyright issues. Third, OA journals have a potential economic interest in publishing a paper with a potentially impaired assessment of its scientific content [19].

Unfortunately, not all OA journals seriously ensure the quality and integrity of research because of the increasing number of predatory journals that are only searching for financial gain. The rush to publish has raised predatory journals that exploit researchers with quick publishing promises, fee demands, and no peer review, thereby eroding scientific integrity [20]. Their aggressive marketing and lenient acceptance pull authors, compromising the credibility of medical science, and potentially misleading readers such as patients who trust this research [21]. Differentiating earnest companies that aim to legitimately establish scientific journals from truly predatory ones is particularly difficult [22]. Furthermore, journals cannot ensure quality control on the use of artificial intelligence (AI) tools, such as natural language processing (NLP) technologies used by authors to enhance academic writing. This brings potential ethical risks and affects the authenticity and trustworthiness of academic contributions [23]. Identifying predatory journals remains challenging despite the proposed criteria, as some new journals use dubious tactics to attract submissions. According to Beall’s list [24], created in 2008 and subsequently closed in 2017 [25], there are some criteria to identify predatory journals, including: the scope of interest appears to include non-biomedical subjects alongside biomedical topics, the website often contains spelling and grammar errors, the homepage language targets authors, description of the manuscript handling process is lacking, manuscripts are requested to be submitted via email, lack of retraction policy, an often very low APC, and the journal retains copyright of published research or fails to mention copyright.

A further item corresponds to massive invitations to authors to publish, based on previous authors publications or congress presentations, as this is quite usual in predatory journals and is usually associated with OA. The invitations can be annoying, and the vast majority come from predatory journals, most of which claim to be OA and to provide peer review [26]. Researchers early in their career might be particularly vulnerable to these invitations, and academic institutions might also consider guidance or policy initiatives to safeguard the interests of their research community and trainees [24–26].

Generally, OA of all variants represents a wonderful opportunity for authors to increase their visibility and ranking; however, owing to the increasing number of OA journals, this represents a true cascade that should be channeled and regulated [27]. There are some possible solutions to regulate the complicated field of new open access journals, which should be identified. Generally speaking, OA journals should follow the same rules of quality as any scientific journal.

1. OA journals should have an established Editorial Board with recognized experts in a specific journal field from all over the world.
2. OA journals should have a list of official reviewers and clearly describe their publication vision and review cycle for each submitted research paper.
3. Every OA journal should have or aspire to have an official impact factor to be indexed in the Root Society for Indexing and Impact Factor Service with a valid ISSN.
4. Every OA journal should have published at least ten continuous articles to be considered for registration.
5. Every OA scientific journal should be in a triage phase for at least 12 months before being registered.
6. Developing countries should have access to existing publishing platforms, which would increase the access of researchers from these countries to important information including predatory journals.
